# Current concepts and perspectives for articular cartilage regeneration

**DOI:** 10.1186/s40634-022-00498-4

**Published:** 2022-07-01

**Authors:** Livia Roseti, Brunella Grigolo

**Affiliations:** IRCCS Istituto Ortopedico Rizzoli Bologna, Bologna, Italy

**Keywords:** Cartilage regeneration, Tissue engineering, Scaffolds, Growth factors, Osteochondral defects bioprinting

## Abstract

Articular cartilage injuries are common in the population. The increment in the elderly people and active life results in an increasing demand for new technologies and good outcomes to satisfy longer and healthier life expectancies. However, because of cartilage's low regenerative capacity, finding an efficacious treatment is still challenging for orthopedics.

Since the pioneering studies based on autologous cell transplantation, regenerative medicine has opened new approaches for cartilage lesion treatment.

Tissue engineering combines cells, biomaterials, and biological factors to regenerate damaged tissues, overcoming conventional therapeutic strategies. Cells synthesize matrix structural components, maintain tissue homeostasis by modulating metabolic, inflammatory, and immunologic pathways. Scaffolds are well acknowledged by clinicians in regenerative applications since they provide the appropriate environment for cells, can be easily implanted, reduce surgical morbidity, allow enhanced cell proliferation, maturation, and an efficient and complete integration with surrounding articular cartilage. Growth factors are molecules that facilitate tissue healing and regeneration by stimulating cell signal pathways.

To date, different cell sources and a wide range of natural and synthetic scaffolds have been used both in pre-clinical and clinical studies with the aim to find the suitable solution for recapitulating cartilage microenvironment and inducing the formation of a new tissue with the biochemical and mechanical properties of the native one. Here, we describe the current concepts for articular cartilage regeneration, highlighting the key actors of this process trying to identify the best perspectives.

## Introduction

Articular cartilage covers the bony ends of diarthrodial joints; it is a smooth thin hyaline tissue with friction-reducing and load-bearing functions. It lacks blood vessels, lymphatics, and nerves [1]. As for connective tissues, an extracellular matrix (ECM) composed of water, collagens, and proteoglycans surrounds the cells. Chondrocytes are mature secretory scarcely distributed cells located in spaces termed lacunae. They are spread among superficial, middle, deep, and calcified four zones. Those zones display different cell shapes, secretory patterns, and fiber orientation [2]. The layer of bone below the hyaline cartilage is known as subchondral bone. It has a structural and mechanical function (shock absorber) and can be involved in the etiology or effects of cartilage damages or diseases [3].

Chondral (affecting articular cartilage) and osteochondral (affecting cartilage and the underlying bone) lesions are very common and can appear at any age [4, 5]. In the young population, they are most of traumatic origin (sport or accident), often with ligament and meniscal injuries. Some conditions like osteochondritis dissecans may lead to articular surface disruption and release of intra-articular bodies composed of cartilage or cartilage and bones [6]. In the elderly population, lesions are associated with rheumatic diseases (i.e., Osteoarthritis-OA) or derived from wear and tear due to excessive use (occupational injury) or age [7, 8].

In general, repair of full-thickness cartilage lesions mainly depend on the patient’s age, defect size, and location. Small full-thickness defects may adjust through the formation of hyaline cartilage. In contrast, large osteochondral defects repair by forming a scar, fibrous tissue, or fibrocartilage in which the predominant component is collagen type I [2, 9].

This kind of cartilage could be functionally active for a short period, but the tissue does not present the mechanical and strength characteristics of normal cartilage, thus not ensuring the healing of the defect nor the symptom remission. This situation could favor, over time, the development of OA [10].

Conventional cartilage treating procedures are palliative and reparative. Palliative strategies usually represent the first-line treatment to decrease symptoms without addressing the causes. Reparative approaches are abrasions drilling and microfracture. They include a subchondral bone penetration allowing stem cells to migrate from bone marrow to the injury site and form, as explained before, mostly a tissue with fibrous features.

Regenerative methods have emerged as an alternative to tissue repair or replacement to regrow or restore diseased cells, tissues, or organs. Osteochondral grafting represents a possible solution for creating a hyaline-like tissue in the affected area. However, it has shown some drawbacks like donor site morbidity, graft failure (autograft), or possible disease transmission (allograft) [11]. Lately, different therapeutic solutions in cartilage regenerative medicine have emerged.

With this manuscript, we would like to report the main therapeutic strategies in this field, focusing on recent approaches in tissue engineering and particularly on up-to-date knowledge’s on cells, growth factors, and scaffolds.

### Tissue engineering

Tissue engineering has represented a new fascinating, and innovative approach to regenerating articular tissue [12]. As reported above, this therapeutic strategy involves the use of cells, scaffolds, and growth factors (GF), trying to mimic the complex three-dimensional microenvironment of the joint that requires the interaction between these different components. Reproducing such complexity is critical, and many issues are still open concerning the ideal cell population, the use of GF and the suitable scaffolds [1, 13].

### Cells

Cells can be administered as therapeutic agents to rebuild damaged cartilage in joints. The leading cell types used in treating chondral and osteochondral defects are chondrocytes and mesenchymal stromal cells from various sources [14]. The technique requires that cells are isolated and then expanded ex vivo in a monolayer culture before the implant. In terms of legislation, expanded cells belong to Advanced Therapy Medicinal Products (ATMPs) and must follow specific rules already encoded for conventional drugs and known as Good Manufacturing Practices (GMPs). GMPs entail the standardization and control of medicinal manufacturing, ensuring their safety and reducing contaminations [15].

### Autologous chondrocyte implantation

Autologous Chondrocyte Implantation (ACI) is a two-step procedure that has been used in the clinic for many years [16]. In the original ACI technique (first-generation technique), the first step consisted of surgically removing small biopsies of normal cartilage from non-weight-bearing areas of the knee. Chondrocytes were then enzymatically isolated from the biopsies, expanded ex vivo in monolayer culture condition, and, after several weeks, harvested as a cell suspension. In the second step, surgeons injected the cell suspension under a periosteal flap harvested from the proximal medial tibia and previously sutured over the cartilage. Chondrocyte expansion was deemed necessary due to cartilage cell scarcity [17].

ACI that uses suspended cultured chondrocytes with a covering of collagen type I/III membrane is considered a second-generation. Third-generation ACI comprises those procedures that deliver autologous cultured chondrocytes using cell carriers or cell-seeded scaffolds. These second and third generation modifications are also known as autologous chondrocyte implantation using collagen membrane (C-ACI), membrane-associated autologous chondrocyte implantation (MACI), and scaffold-based ACI [13, 18]. These procedures were introduced in the clinical practice one decade ago, showing similar results while at the same time overcoming most of the concerns related to the first-generation ACI. The use of scaffolds to create a cartilage-like tissue in a three-dimensional culture system allows for the optimization of the procedure from both the biological and surgical points of view [19, 20].

Although good clinical radiological and histological outcomes of the different ACI procedures, one of the main drawbacks is the need for a cell expansion phase which is long-lasting, complicated, and expensive primarily due to GMPs requirements. Moreover, the need for two hospitalizations increases the risk for the patients and the costs for the public health system. For all these reasons, investigations have been moving towards different cell populations as reported below [9, 21, 22].

### Mesenchymal stromal cells (MSC)

Stromal cells from various sources are currently available for cartilage regeneration. This is due to their ability to proliferate in culture and directionally differentiate by synthesizing structural and functional hyaline ECM molecules. Moreover, they can release many anti-inflammatory, anti-apoptotic, and immuno-modulatory factors favoring the healing process [23]. Many studies have reported benefits in treating cartilage injuries with adult bone marrow-derived MSC [23]. Adipose-derived stem cells (ASC) have also drawn attention for their analogy with bone marrow ones, but with easier harvesting, a higher cell density, and proliferation. Other sources of stem cells investigated for cartilage repair include muscle, synovial membrane, trabecular bone, dermis, blood, umbilical cord blood, and periosteum [23]. Although various successful applications in cartilage regeneration, several problems remain, like stem cell heterogeneity and premature differentiation during in vitro expansion [24].

Induced pluripotent stem cells (iPSCs) have a promising potential for cartilage regeneration. Besides, they allow overcoming limitations associated with current cell sources since large numbers of cells can derive from small starting populations. However, issues related to genomic modifications still need addressing [25].

Genetically modified cells showed the ability to potentiate cartilage regeneration. Transfected genes inducing chondrogenic differentiation, synthesis of a hyaline matrix, and release of pro-inflammatory factors differentiation are feasible. Gene transfection may be systemic or local, ex vivo or in vivo. Because cartilage injuries are not life-threatening, it is critical to ensure a safe procedure [26].

MSC, as a pure cell population, require the selective elimination of cells that do not express their typical markers. Recently, new insight turned into the role of the surrounding MSC microenvironment (or “niche”) that also encloses ECM, accessory cells, adhesion molecules, growth factors, cytokines, and chemokines. Stem cell activity is not only the expression of intrinsic capabilities but also the result of the interactions with the “niche”. It is the whole “niche” that can support the healing process. No cell selection and expansion in the laboratory are necessary, and a single operative procedure is effective [27–29].

In recent years, also articular cartilage regeneration research moved towards the use of the stem cell “niche” in the form of concentrates such as Bone Marrow Concentrate (BMC) and Stromal Vascular Fraction (SVF) from adipose tissue. Both concentrates are obtained with minimal manipulation (no expansion in culture) and provide a less invasive (one step-surgery) and less expensive (no GMPs) alternative to cultured cells.

Our laboratory investigated the behavior of BMC cells within a hyaluronan-based scaffold. Histological immunohistochemical and molecular results showed the formation of a cartilage-like ECM [30]. We also evaluated BMC chondrogenic and osteogenic potential on a bi-layered scaffold mimicking the osteochondral compartment (collagen and hydroxyapatite). The obtained data demonstrated the ability to reproduce the native osteochondral compartment by generating two separated cartilage and bone zones [31, 32].

SVF obtained from lipoaspirate contains several cell types like ASCs, ECM fibroblasts, and white and red blood cells. After washing passages, the obtained SVF can be combined with scaffold and soluble factors and administered into the joint. Compared to BMC, SVF ensure easier accessibility and the availability of an increased number of stem cells per gram of tissue [33].

### Cell free products

In the early stages, it seems that the ability of MSC to differentiate into various cell types played the main therapeutic effect. Later, it emerged that their capacity to release some GF and chemokines play a role (secretome). MSCs secrete bioactive molecules inhibiting apoptosis and the formation of fibrosis or scarring at the injury site; stimulate angiogenesis and blood supply, and mitosis of tissue-specific progenitors. They also secrete immunomodulatory agents that deactivate the T cells surveillance and chronic inflammatory processes. Therefore, the secretome use for tissue regeneration increased, based on its composition of trophic factors (chemokines, cytokines, hormones, and lipid mediators) with paracrine effects on the cells of the local microenvironment [34].

The soluble factors of the secretome can initiate regenerative signaling events also without the use of cells. The therapeutic effect of this biological product in musculoskeletal diseases is a frontier of regenerative medicine. The secretome could overcome the negative aspects of cell use and help concentrate paracrine factors at physiological levels at the injury site.

Although many studies provide strong evidence for the potency of MSC-secreted factors in mediating tissue repair and regeneration, the precise mechanisms of action are still not fully understood [35].

The paracrine action of MSC is not limited to the production of soluble factors but also of many extracellular vesicles (EVs). EVs [36] are involved in intercellular communication by releasing mRNAs and proteins. Besides, they have anti-apoptotic, antifibrotic, pro-angiogenic, and anti-inflammatory effects. EVs released from tissue-damaged cells can re-program stem cells' phenotype by releasing specific mRNAs or microRNAs. EVs produced by circulation-recruited or resident MSCs can re-program tissue-damaged cells by inducing de-differentiation, production of soluble paracrine mediators, and initiation of the cell cycle of these cells, thus promoting tissue regeneration [37].

### Growth factors

Biologic agents represent an emerging treatment for several musculo-skeletal pathologies. These agents are mainly represented by natural GF and anti-inflammatory mediators that can accelerate tissue healing and regeneration. They can act through various mechanisms, including matrix synthesis and remodeling, cell recruitment and modulation of inflammatory markers and metalloproteinases.

Moreover, GF may influence protein synthesis and cellular interactions, controlling stem cell differentiation. Bone Morphogenic Protein-2 (BMP-2) regulates osteogenesis, Vascular Endothelial Growth Factor (VEGF) angiogenesis, and Transforming Growth Factor-β1 (TGF-β1) chondrogenesis. The possible role played by GF in pian reduction and tissue regeneration has generated a growing interest in their possible therapeutic use in patients with musculo-skeletal injuries.

Recently, discoveries, combined with knowledge of the importance and role of growth factors for tissue engineering, have been further developed and deepened [38].GF facilitate and promote the regeneration of new tissues by interaction with specific transmembrane receptors and regulating protein synthesis within cells. Binding to the specific growth factor receptor specifically stimulates cell signal transduction pathways that trigger cell migration, survival, adhesion, proliferation, growth, and differentiation.

Although GF have great potential to stimulate cartilage repair, only a limited number of treatments have been approved by government regulatory agencies for clinical use [39].

Platelet-rich plasma (PRP) represents an economical source for obtaining many GF in physiological proportions and has already been widely applied in various fields of medicine for its property of promoting tissue regeneration [12, 40]. PRP can be defined as a blood derivative product in which platelets are present in high concentration. Platelets have demonstrated regenerative properties because they are rich in important GF.

In particular, α platelet granules contain and release numerous GF including PDGF, TGF-β1, VEGF, Epidermal Growth factor (EGF), Fibroblast Growth Factor (FGF) and Insulin-like Growth Factor (IGF).

In recent years, PRP has achieved great success in clinical practice, thanks to its safety and simply preparation technique, which allows exploiting its biologically active content.

PRP has been used successfully in several surgical techniques, obtaining good results in association with microfractures or scaffolds for the treatment of cartilage lesions [41]. The most significant evidence on PRP is instead for its intra-articular use in the treatment of osteoarthritis, especially in the knee. Despite this, the most suitable type of PRP remains debated, with different preparation methods available that can give products with different composition and properties [42].

### Scaffolds

Scaffolds are support sustaining three-dimensional (3D) tissue development. They differ in material composition, structure, and status. An ideal scaffold should be biomimetic, biocompatible, biodegradable, and non-immunogenic; induce cell attachment, growth, and differentiation. Once implanted, it should integrate into the lesion site and support the healing process. It should also be easy to handle by surgeons, and cost-effective Scaffolds for cartilage regeneration may be natural, or synthetic [43].

Natural materials possess high biocompatibility and bioactivity. However, show poor mechanical stability because of their rapid hydrolysis. A list of the most known natural materials with the principal advantages and disadvantages is reported in Table 1.

**Table 1:** Natural materials for cartilage tissue engineering. Advantages and disadvantages

Natural origin scaffolds	Advantages	Disadvantages
Hyaluronic acid	Anionic, non-sulfated glycosaminoglycan (GAG) is present in cartilage ECM. Supports cell attachment through surface receptors like CD44ECM	Poor mechanical properties, rapid degradation
Chondroitin sulfate	Sulfated GAG is present in cartilage ECM with anti- inflammatory activity, and a role in cell signaling. Easy to be functionalized	Poor mechanical properties, rapid degradation
Alginate	Negatively charged polysaccharide extracted from brown algae and bacterial sources. High functionality, fast cross-linking, low cost, injectable for bioprinting, structural similarity to GAGs	Poor mechanical strength, low cell- matrix interaction, varying levels of purity due to source variability, immunogenicity
Agarose	A marine polysaccharide obtained from seaweed. It presents excellent biocompatibility, good stiffness and viscoelasticity. High functionality, thermoreversible gelation, low cost, structural similarity to GAGs	Limited mechanical performance, low bioactivity, poor cell attachment
Chitosan	An amino polysaccharide polymer derived from chitin and the wastes of the seafood industry. Biocompatible and biodegradable. It possesses antibacterial ability	Poor water solubility in physiological conditions, potential allergenic risks, inferior mechanical properties, low cell adhesiveness, and potential allergenic reactions due to its origin
Gellan gum	A linear negatively charged polysaccharide produced by the Sphingononas group bacteria; pH and temperature responsiveness, structural similarity to GAGs	Weak mechanical strength, poor stability, low bioactivity, relatively high gelation temperature, small temperature window
Collagen	The main protein component in natural cartilage, displays great biocompatibility and biodegradation without causing inflammation	Poor mechanical properties, potential of immunogenicity, high cost, limited sterilizability
Gelatin	A derivative of collagen by partial hydrolysis with much lower antigenicity Biologically active for cellular interaction, low immunogenicity in comparison to collagen, ease of processing and functionalization	Poor mechanical properties, rapid degradation, low thermal stability
Silk fibroin	The major protein component of natural silk. High mechanical strength, low immunogenicity, morphologic flexibility, good sterilizability, usable for cartilage bioprinting, easily available, biocompatible, biodegradable	Source variability, low biodegradability
Fibrin	Fibrin is a blood protein, well known for its role in clot formation, justifying its use in clinical practice as a hemostatic or a sealant agent. Hydrogels can be prepared from fibrinogen by the enzymatic treatment of thrombin; the advantages are excellent biocompatibility and biodegradability	Weak mechanical properties
Cellulose	One of many polymers found in nature, may enter the composition of carboxymethyl cellulose, and in turn, hydrogel by specific processes	Low integration. No degradability

Synthetic polymers like poly(ethylene glycol) (PEG), polycaprolactone (PCL), polylactic acid (PLA), polyurethane, poly(glycolic acid) (PGA), polyethersulfone (PES), and polysulfone provide cell attachment, and good mechanical, physical, and chemical properties. Moreover, the mechanical properties and degradation time can be controlled by combining them as copolymers or blends. Disadvantages consist of the lack of biological properties and the host organism's side effects in response to metabolite production, mainly concerning acids that can be toxic or induce an inflammatory response [43].

Hybrid scaffolds, such as a combination of collagen-chitosan- PLA, merge the advantages of synthetic and natural materials, allowing biocompatible membranes with defined mechanical properties and tunable degradation necessary for cartilage regeneration [43].

Studies highlighted the in vitro and in vivo interaction of cells with scaffolds [44]. Our group had the opportunity to evaluate some natural scaffolds based on collagen or hyaluronan. We highlighted that scaffold presence allows the re-creation of physiological-like conditions whereby cells interact with the biomaterial and produce a new ECM by the secretion of anabolic, anti-inflammatory, and anti-apoptotic factors [45–50].

A challenge in the design and fabrication of scaffolds is the reproduction of the osteochondral compartment. To this end, composite bilayer or gradient scaffolds mimicking the osteochondral tissue have been developed and evaluated in association with cells. The data obtained demonstrated cell ability to zonally interact and reproduce the native osteochondral compartment by generating separated cartilage- and bone-like zones [5].

Another challenge is the cell seeding onto the scaffold. Conventional method involves the manual/static or the automated/dynamic seeding of cells onto previously fabricated scaffolds. The static seeding allows an uneven cell distribution into the width of the biomaterial. The dynamic seeding carried out with bioreactors (for instance perfusion) favor a more homogenous cell distribution [24]. The recent approach of bioprinting foresees that cells and biomaterial are released together in order to produce a construct. Such options allow a better cell encapsulation and spatial distribution [51].

### Future directions

In the next decades we will assist to important steps forwards the repair of articular cartilage lesions. The use of iPSCs and or stem cell derivatives such as secretome, EVs could contribute to improve tissue regeneration.

Emerging technologies like Additive Manufacturing three-dimensional (3D) printing should allow for a further improvement of the treatment. 3D printing replicates the damaged tissue shape starting from a patient medical image. It creates scaffolds through the progressive addition of material layer by layer until reaching the desired shape. The technology can mimic cartilage organization, ECM composition, and functional and mechanical properties [52–55].

Indeed, the identification of the ideal cell population, cell-free products, clinical grade growth factors and customized scaffolds could contribute to ameliorate the technique, reducing the time for surgery and enhancing patient recovery.

## Conclusion

Chondral and osteochondral damages remain a tough challenge for clinicians. Tissue engineering-based strategies have proven feasible for cartilage regeneration with good results on patients' quality of life. More research needs to find the best combinations of cells, bioactive factors, and scaffolds. More clinical trials should confirm the obtained results. There is also the demand to develop minimally invasive and cost-effective strategies which do not require long-lasting hospitalization.
